# Supplementary material for: Combination of Tipifarnib and Sunitinib Overcomes Renal Cell Carcinoma Resistance to Tyrosine Kinase Inhibitors via Tumor-Derived Exosome and T Cell Modulation
Source: Cancers (Basel). 2022 Feb 11;14(4):903. doi: 10.3390/cancers14040903 (PMC8870174; doi:10.3390/cancers14040903)
Supplement: Supplementary file 1 [file cancers-14-00903-s001.zip › cancers-1532798-supplementary.pdf]

cancers

Article

Combination of Tipifarnib and Sunitinib Overcomes Renal Cell Carcinoma Resistance to Tyrosine Kinase Inhibitors via Exosome and T Cell Modulation.

Jacob W. Greenberg, Hogyoung Kim, Miae Ahn, Ahmed A. Moustafa, He Zhou, Pedro C. Barata, A. Hamid Boulares, Asim B. Abdel-Mageed and Louis S. Krane

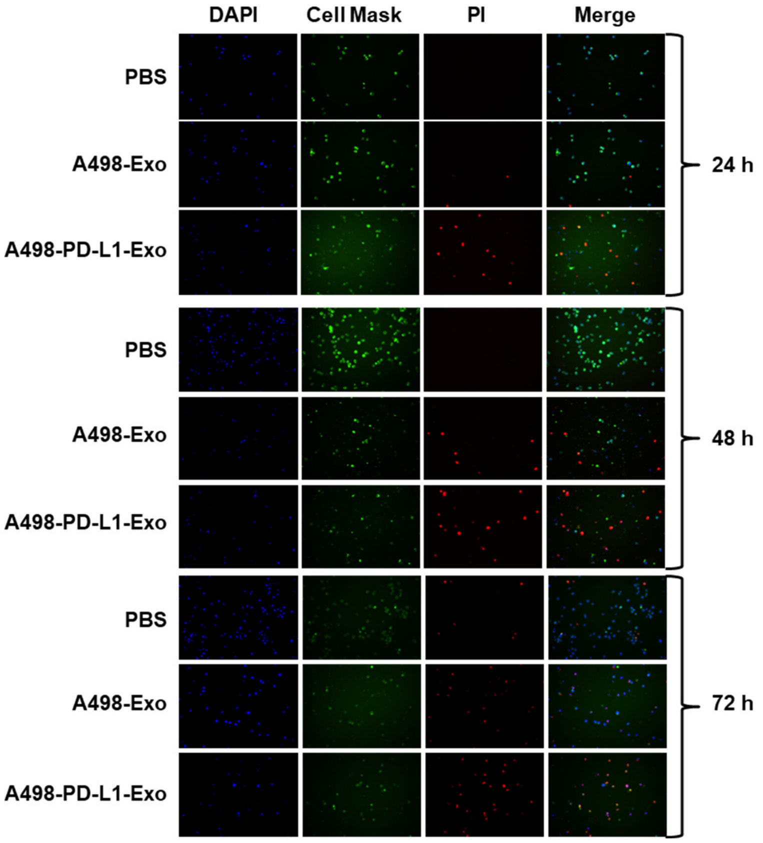

Figure S1. Representative composite images show morphological changes of Jurkat T cells detected with triple staining of Hoechst 33342, Cell Mask™ Green plasma membrane stain, and PI. Cells were treated with exosomes a difference time point, and imaged by fluorescence microscope.

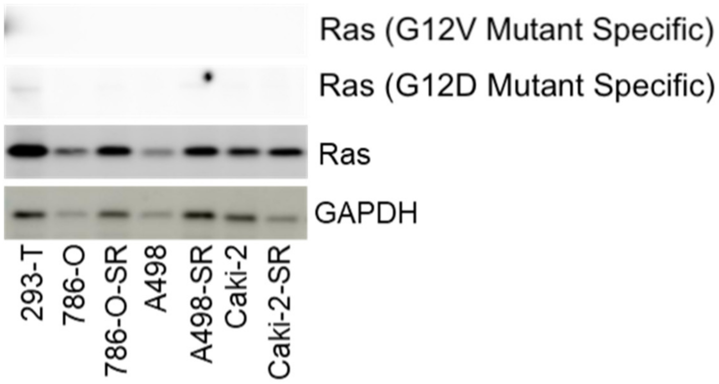

Figure S2. The expression of H-Ras G12V, H-Ras G12D and total Ras in SS, or SR RCC cell lines. Western blot analysis of extracts from various RCC cell lines using Ras (G12V Mutant Specific) (D2H12) Rabbit mAb (upper), Ras (D2C1) Rabbit mAb #8955 (middle), and GAPDH sc-s7725 (lower). Full blots are presented in Supplementary Figure S8.

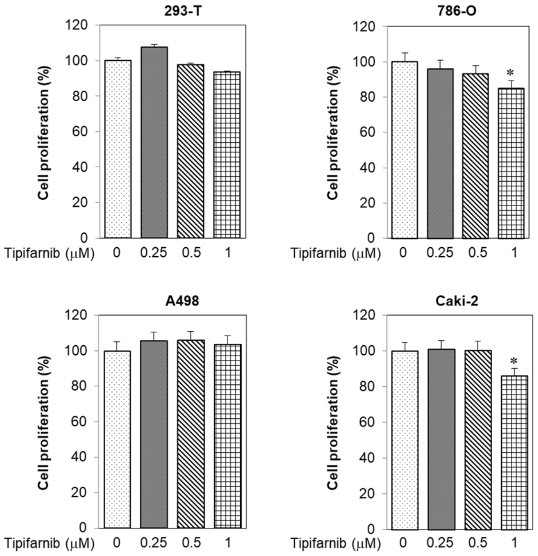

Figure S3. Effect of tipifarnib on cell viability. 293-T, 786-O, A498 and Caki-2 cells were treated with vehicle (0; DMSO) or a dose dependent manner of tipifarnib for 48 h. Viable cells were measured by MTT assay (*n*=3). Data were analyzed by one-way ANOVA followed by Bonferroni post-tests. *$p<0.05$, vs. control.

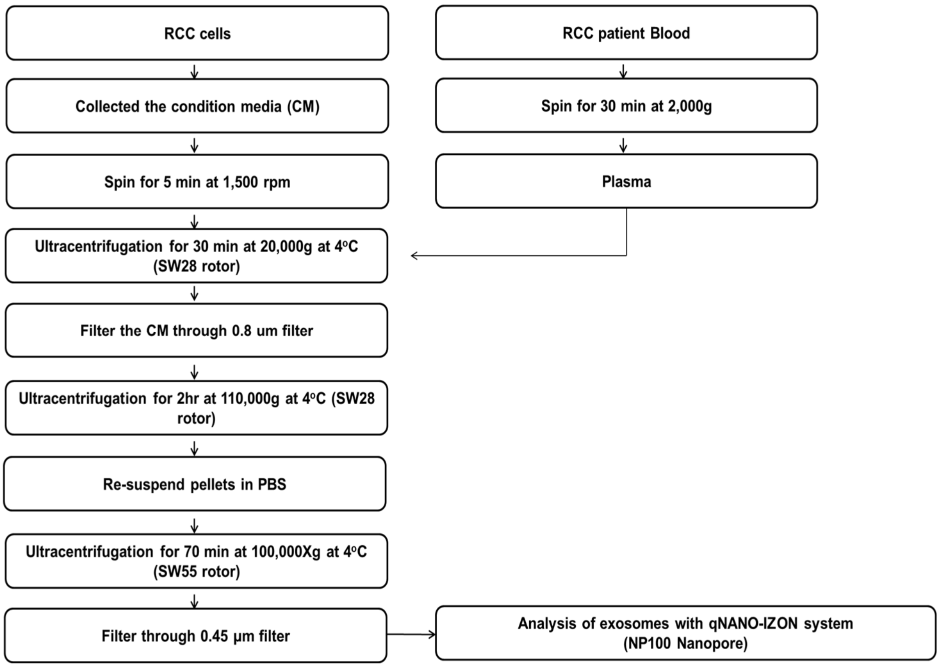

Figure S4. Flowchart of isolation and analysis of the extracellular vesicles (EVs). Purification and analysis of EVs including exosomes in the conditioned media (CM) of RCC cells treated with DMSO (vehicle) or tipifarnib (0.5 μM) were performed according to the depicted flow chart. Following differential ultracentrifugation, MVs were prepared by filtration through 0.8 μm and 0.45 μm filters, respectively, and analyzed by qNano-IZON system using NP100 (size range: 50-250 nm) nanopore, respectively.

		Mode	StDev	Mean
293-T	DMSO	109	19.5	104.5
	0.5 μM Tipifarnib	108.5	17.7	102.5
		Mode	StDev	Mean
786-O	DMSO	127	37.9	101
	0.5 μM Tipifarnib	137.5	44.35	108
		Mode	StDev	Mean
A498	DMSO	113.5	40.25	88.5
	0.5 μM Tipifarnib	115	47.05	89
		Mode	StDev	Mean
Caki-2	DMSO	103.5	29.9	86.5
	0.5 μM Tipifarnib	111.0	35.2	88.5

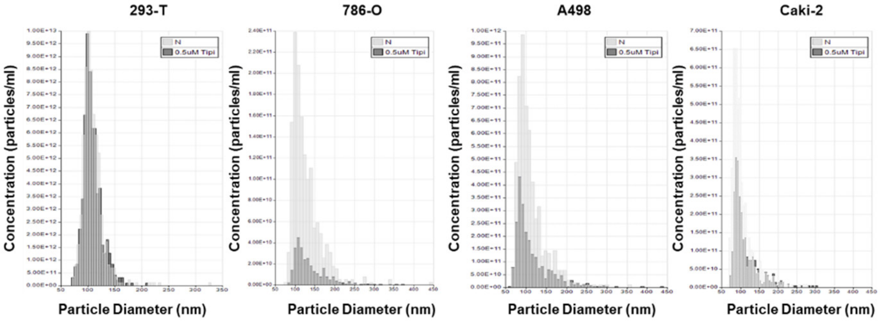

Figure S5. Effect of tipifarnib on the diameter of SS-RCC cell-secreted EVs. 293-T, 786-O, A498 or Caki-2 cells were treated with tipifarnib (0.5 μM) or DMSO at different time intervals and particle diameter and diameter mode of exosomes were measured with qNano-IZON system. There was no significant difference in the exosome diameter or diameter mode of exosomes harvested from tipifarnib-treated or DMSO-treated 293-T, 786-O, A498 or Caki-2 cells.

		Mode	StDev	Mean
786-O-SR	DMSO	108	32.25	89
	0.5 µM Tipifarnib	113.0	45.6	81.0
A498-SR	DMSO	102.5	34.4	70.5
	0.5 µM Tipifarnib	105.0	35.8	74.5
Caki-2-SR	DMSO	105.7	37.5	78.7
	0.5 µM Tipifarnib	104.0	37.1	76.5

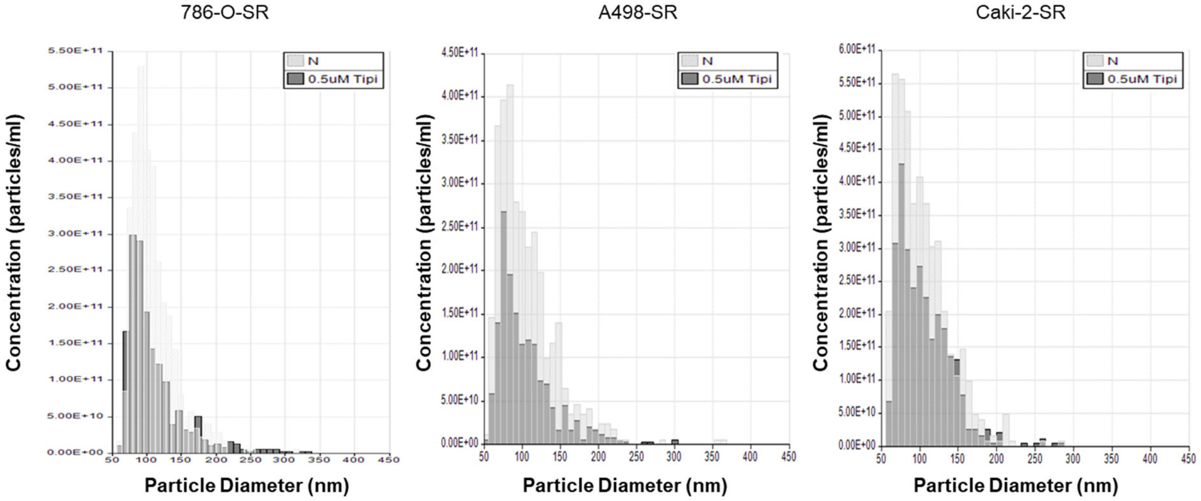

Figure S6. Effect of tipifarnib on the diameter of SR-RCC cell-secreted EVs. 786-O-SR, A498-SR, or Caki-2-SR cells were treated with tipifarnib (0.5 µM) or DMSO at different time intervals and particle diameter and diameter mode of exosomes were measured with qNano-IZON system. There was no significant difference in the exosome diameter or diameter mode of exosomes harvested from tipifarnib-treated or DMSO-treated 786-O-SR, A498-SR or Caki-2-SR cells.

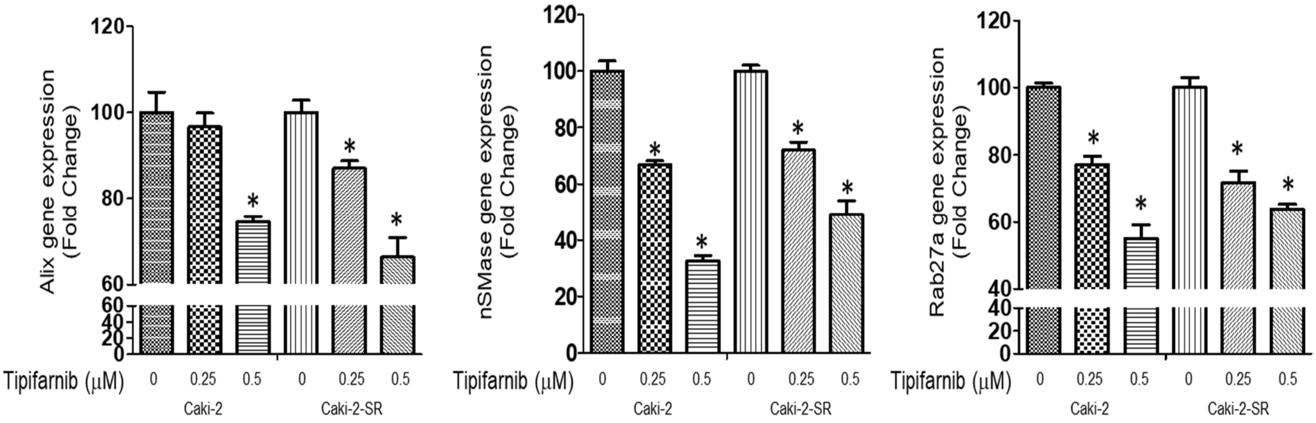

Figure S7. Tipifarnib effects on Alix, Rab27a, and nSMase2 mRNA expression in RCC cells. Levels of Alix, Rab27a and nSMase2 mRNA after tipifarnib exposure, measured by real-time reverse transcription-PCR. cDNA was synthesized from RNA samples from control-and tipifarnib-exposed cells. Alix, Rab27a and nSMase2 expression values are relative to the levels of GADPH. *p<0.05, vs. control.

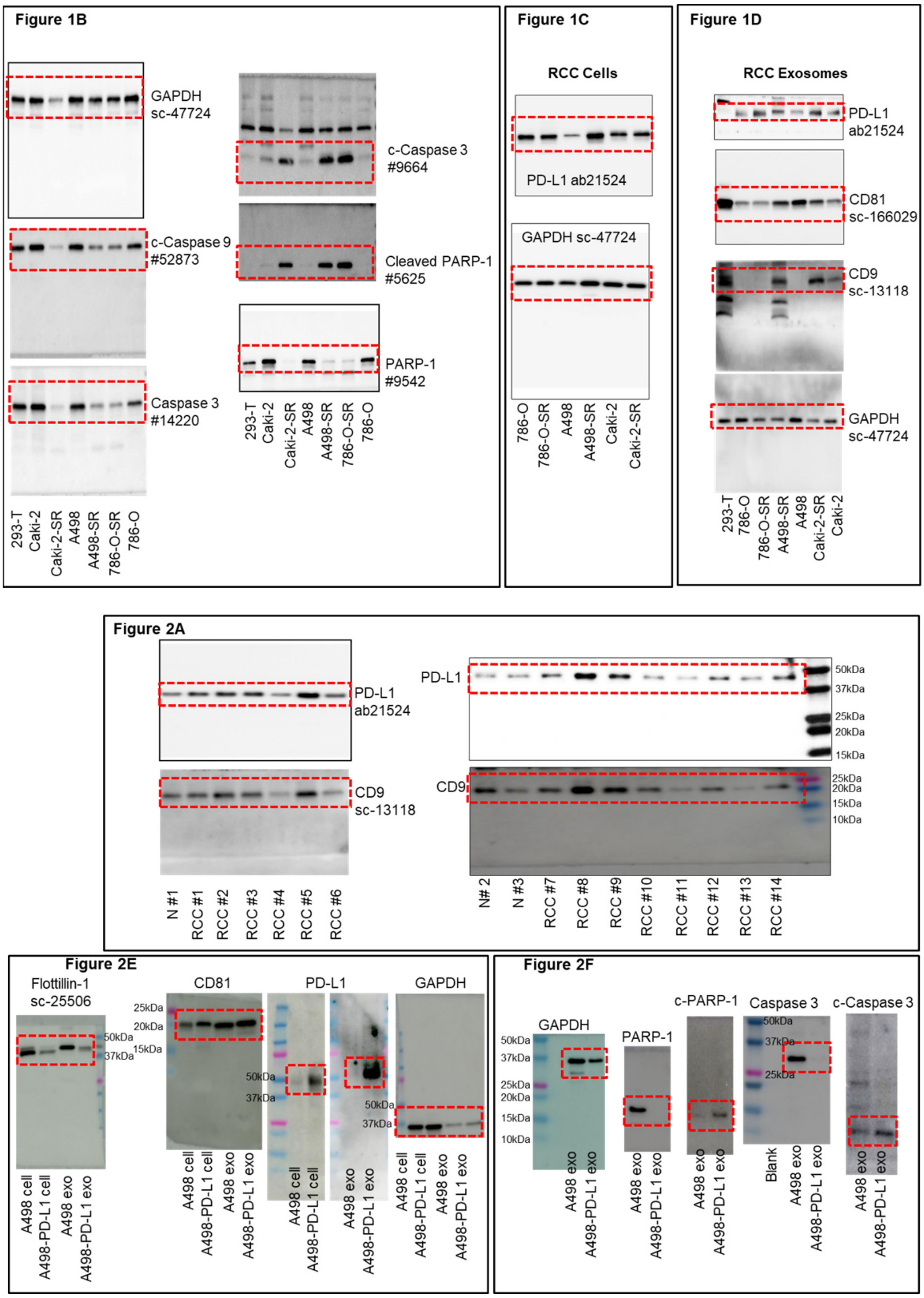
Figure 1B
GAPDH sc-47724
c-Caspase 3 #9664
c-Caspase 9 #52873
Cleaved PARP-1 #5625
PARP-1 #9542
Caspase 3 #14220
293-T
Caki-2
Caki-2-SR
A498
A498-SR
786-O-SR
786-O
Figure 1C
RCC Cells
PD-L1 ab21524
GAPDH sc-47724
786-O
786-O-SR
A498
A498-SR
Caki-2
Caki-2-SR
Figure 1D
RCC Exosomes
PD-L1 ab21524
CD81 sc-166029
CD9 sc-13118
GAPDH sc-47724
293-T
786-O
786-O-SR
A498-SR
A498
Caki-2-SR
Caki-2
Figure 2A
PD-L1 ab21524
CD9 sc-13118
N #1
RCC #1
RCC #2
RCC #3
RCC #4
RCC #5
RCC #6
PD-L1
CD9
50kDa
37kDa
25kDa
20kDa
15kDa
10kDa
N# 2
N #3
RCC #7
RCC #8
RCC #9
RCC #10
RCC #11
RCC #12
RCC #13
RCC #14
Figure 2E
Flottillin-1 sc-25506
CD81
PD-L1
GAPDH
A498 cell
A498-PD-L1 cell
A498 exo
A498-PD-L1 exo
Figure 2F
GAPDH
PARP-1
c-PARP-1
Caspase 3
c-Caspase 3
Blank
A498 exo
A498-PD-L1 exo

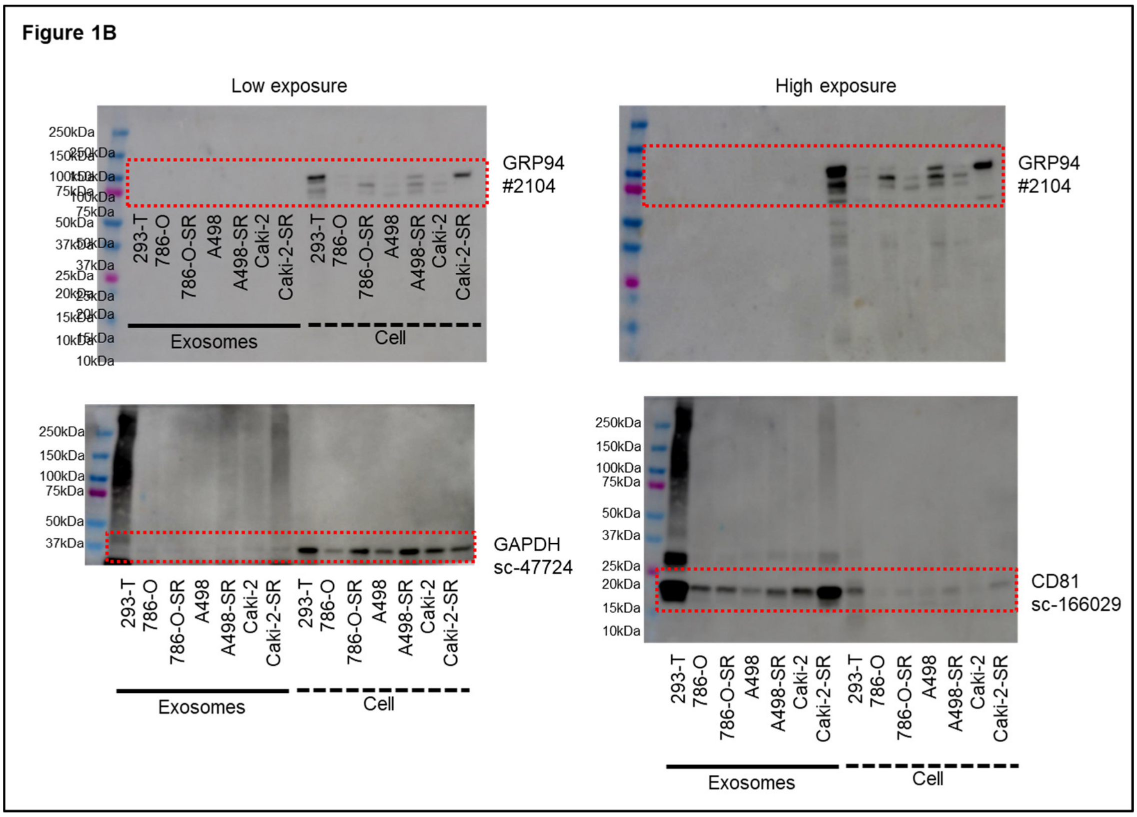
Figure 1B
Low exposure
High exposure
GRP94
#2104
GAPDH
sc-47724
CD81
sc-166029
293-T
786-O
786-O-SR
A498
A498-SR
Caki-2
Caki-2-SR
Exosomes
Cell
250kDa
150kDa
100kDa
75kDa
50kDa
37kDa
25kDa
20kDa
15kDa
10kDa

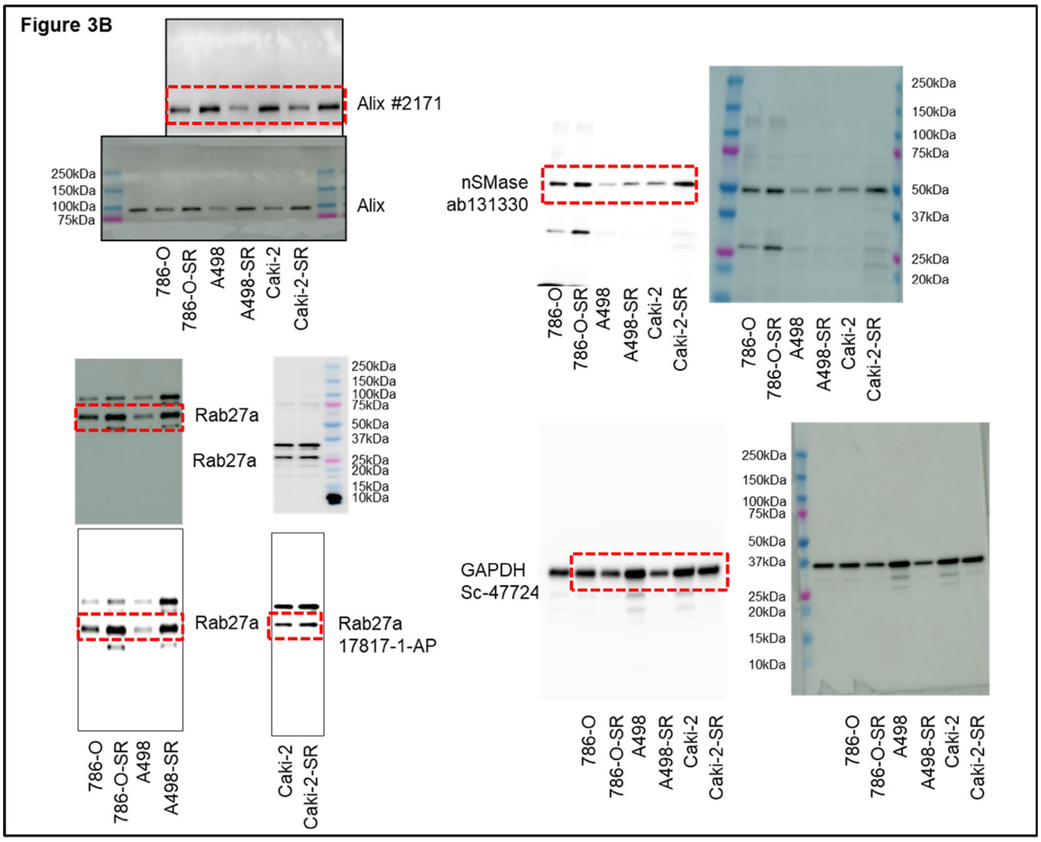
Figure 3B
Alix #2171
Alix
nSMase
ab131330
Rab27a
Rab27a
17817-1-AP
GAPDH
Sc-47724
786-O
786-O-SR
A498
A498-SR
Caki-2
Caki-2-SR
250kDa
150kDa
100kDa
75kDa
50kDa
37kDa
25kDa
20kDa
15kDa
10kDa

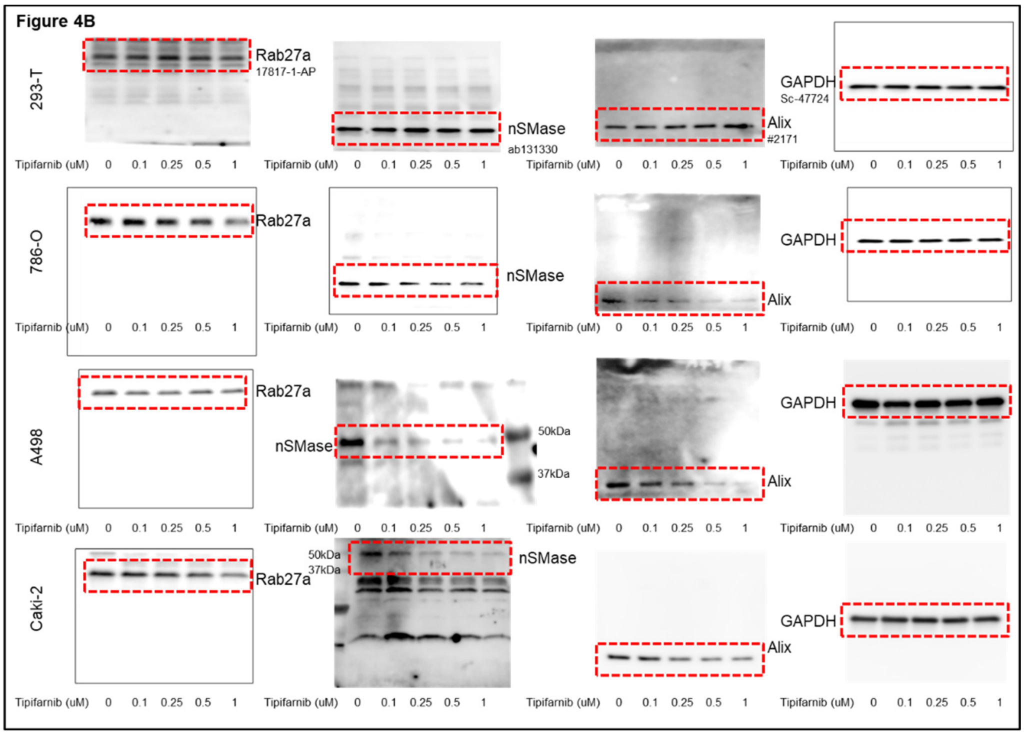
Figure 4B
293-T
786-O
A498
Caki-2
Rab27a
17817-1-AP
nSMase
ab131330
Alix
#2171
GAPDH
Sc-47724
50kDa
37kDa
Tipifarnib (uM) 0 0.1 0.25 0.5 1

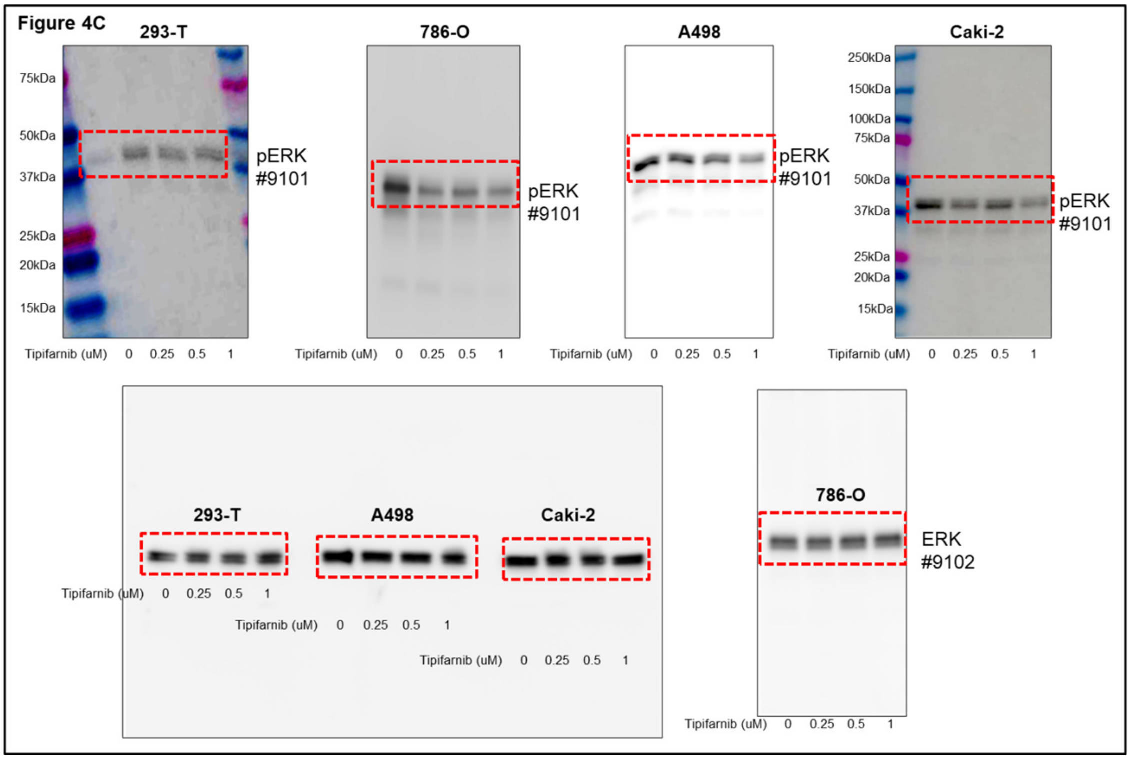
Figure 4C
293-T
786-O
A498
Caki-2
75kDa
50kDa
37kDa
25kDa
20kDa
15kDa
250kDa
150kDa
100kDa
pERK
#9101
ERK
#9102
Tipifarnib (uM) 0 0.25 0.5 1

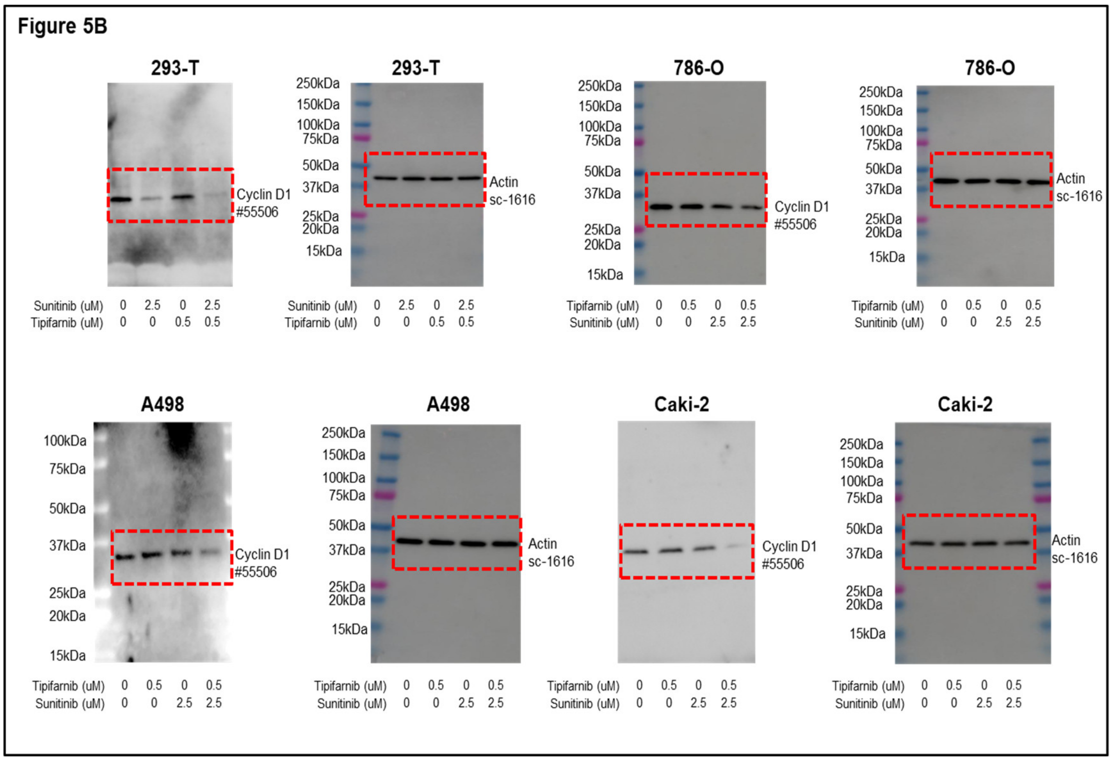

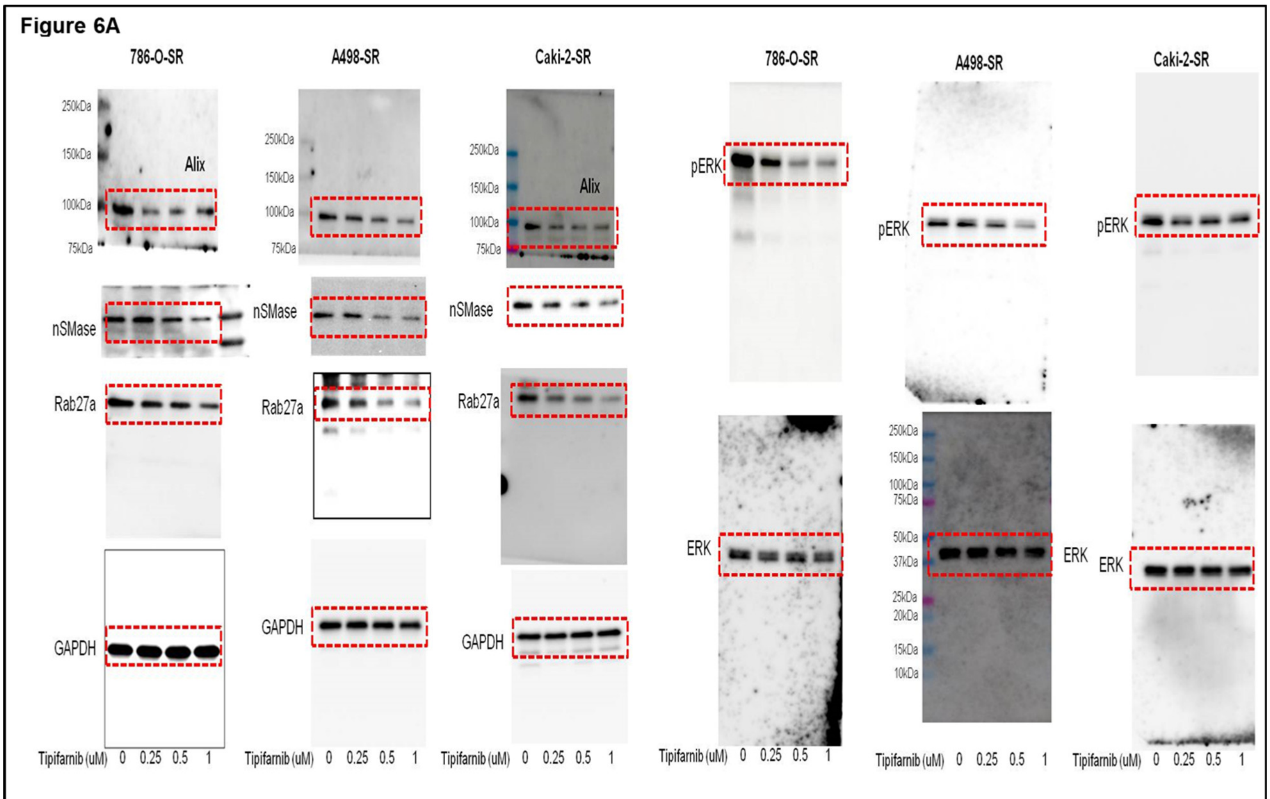

Alix(#2171), nSMase(ab131330), Rabb27a(17817-1-AP), GAPDH(sc-47724), pERK(#9101), and ERK(#9102)

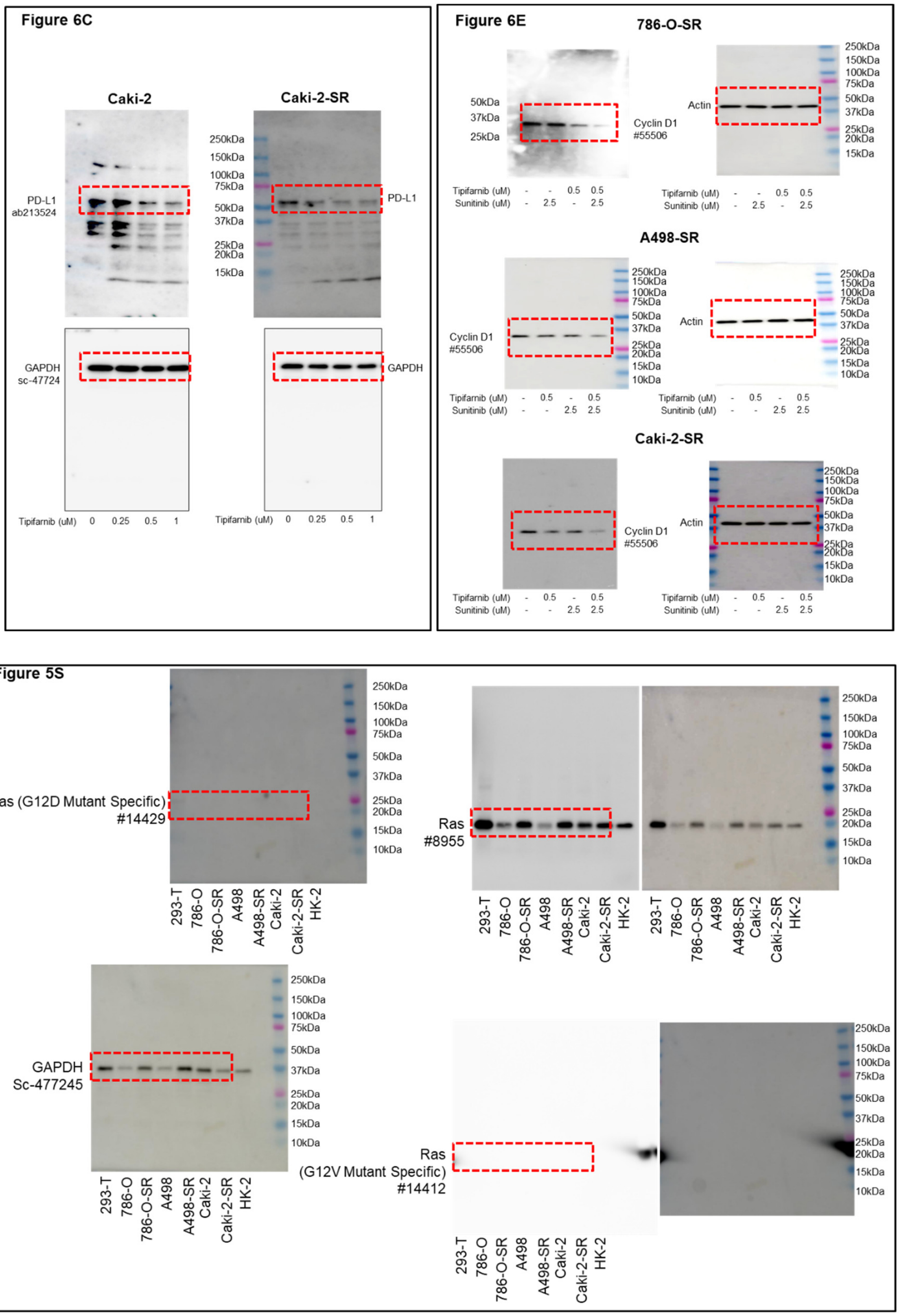

Figure S8. Information of Full blot.

Table S1. Gene, primer orientation, primer sequence (5′ to 3′), and National Center for Biotechnology Information (NCBI) accession number and sequence for primers used in real-time quantitative PCR assays.

Gene	Primer	Sequence (5' to 3')	NCBI Sequence
PDCD6IP	Forward	TGGCTGCAAAGCACTGTATC	NM_013374
	Reverse	AGGGCACGATTGATTTTGTC	
RAB27A	Forward	TGGAGGACCAGAGAGTAGTGAAA	NM_004580.5
	Reverse	GTTTCAAAGTAGGGGATTCCA	
SMPD2	Forward	CAGGAGCTTACCCAGCACAT	NM_003080.3
	Reverse	GTTGAGCACCATGCCACTTA	
GAPDH	Forward	TCCCTGAGCTGAACGGGAAG	NM_001256799.2
	Reverse	GGAGGAGTGGGTGTCGCTGT	

Table S2. Comparison of exosome protein concentration and particle concentration.

	Protein concentration(μg/ml)	Total particles concentration	particles/μg	particles/40 μg
786-O Exo	706.11	1.54E+12	2.18E+09	8.72E+10
786-O-SR Exo	361.67	1.615E+12	2.23E+09	8.93E+10
Caki-2 Exo	476.44	8.14E+11	1.71E+09	6.83E+10
Caki-2-SR Exo	791.67	1.42E+12	1.79E+09	7.17E+10
A498-Exo	623.44	1.875E+12	3.01E+09	1.20E+11
A498-SR Exo	917.22	2.215E+12	2.41E+09	9.66E+10
